# Structural performance of UHPC-columns reinforced with basalt bars under cyclic loading

**DOI:** 10.1038/s41598-025-31316-3

**Published:** 2026-01-10

**Authors:** Taha A. El-Sayed, Muhammed S. Fekry, Hossam E. Ahmed, Ali S. Shanour

**Affiliations:** Department of Civil Engineering, Shoubra Faculty of Engineering, Benha University, Cairo, Egypt

**Keywords:** UHPC, BFRP, Cyclic loading, ABAQUS, Nonlinear FE analysis, Cracking, Deflection, Ductility, Failure mode, Engineering, Materials science

## Abstract

This study investigates the structural performance of ultra-high-performance concrete (**UHPC**) columns reinforced with basalt fiber-reinforced polymer (**BFRP**) bars under cyclic loading. Limited research has been conducted on concrete columns reinforced with basalt bars, particularly under cyclic loading conditions. In this research, six columns were cast, each with a cross-sectional dimension of 150× 150 mm and a height of 1500 mm. Each specimen was supported on a footing measuring 1000 mm in width and 250 mm in height. All specimens were tested under cyclic lateral loading until failure to evaluate their structural performance. A constant axial load of 60 kN was applied and maintained throughout the entire duration of the tests. The 28-day compressive strength of the concrete varied between 75 and 100 MPa, with mixes targeting strengths of 75, 80, and 100 MPa. Experimental results showed that columns reinforced with BFRP bars exhibited improved structural performance compared with those reinforced with conventional steel bars. The BFRP-reinforced columns demonstrated a more controlled failure mode, primarily governed by concrete crushing without reinforcement buckling, and were able to sustain higher loads with narrower crack widths, indicating enhanced deformation capacity and ductility behavior. These results confirm that despite the inherently brittle nature of BFRP reinforcement, its superior tensile strength and bond interaction with the UHPC matrix contributed to an overall improvement in cyclic stability and energy dissipation compared to traditional steel-reinforced specimens. The tensile strength of BFRP bars was nearly twice that of conventional steel reinforcement, with average values of about 1100 MPa for BFRP and approximately 500 MPa for steel. To simulate and validate the structural response of the tested UHPC columns, advanced three-dimensional finite-element models were developed using ABAQUS software. A strong correlation was observed between the numerical and experimental results in terms of initial cracking load, ultimate load capacity, crack propagation, and lateral deflection. The developed nonlinear FE models achieved approximately 85% agreement with the experimental ultimate load capacity.

## Introduction

Ultra-High-Performance Concrete (**UHPC**) has emerged as a promising construction material due to its superior mechanical performance, durability, and long-term sustainability compared to normal-strength concrete (**NSC**)^1,2^. Its exceptional compressive strength and low permeability make it particularly suitable for critical structural components in bridges, marine structures, and high-rise buildings, where strength, durability, and reduced maintenance are essential^1,2^. The optimized particle packing of UHPC allows designers to minimize member cross-sections without compromising strength or stiffness, enhancing the material’s efficiency and service life^3^.

Characterized by a low water-to-cement (**w/c**) ratio (typically between 0.15 and 0.25), UHPC mixes require the inclusion of **superplasticizers (SPs)** to ensure proper dispersion and workability^4^. The incorporation of **silica fume (SF)** and ordinary Portland cement (**OPC**) as major binders contributes to its dense microstructure and resistance to aggressive environmental conditions such as chloride penetration, freeze–thaw cycles, and chemical attack^5–9^. These features result in exceptional durability and structural integrity under severe service environments.

The remarkable mechanical and durability properties of UHPC are attributed to its highly refined microstructure and strong interfacial bonding between the matrix constituents^10,11^. Over the past two decades, UHPC has been increasingly utilized in architectural, marine, and infrastructure projects due to its high strength and longevity^10,12^. Furthermore, its superior durability reduces maintenance demands throughout a structure’s life cycle, enhancing both sustainability and cost efficiency^10,13–15^. Despite these advantages, the adoption of UHPC remains limited by the absence of comprehensive design codes and a need for further experimental validation of its performance under various loading conditions^16–18^.

To fully exploit the potential of UHPC, corrosion-resistant and lightweight reinforcement materials are increasingly sought as alternatives to traditional steel. Fiber-Reinforced Polymer (**FRP**) bars have gained widespread attention due to their high tensile strength, non-corrosive nature, and ease of handling^19–21^. Among FRP types, Basalt Fiber-Reinforced Polymer (**BFRP**) bars offer a favorable balance between mechanical performance and cost efficiency. They provide greater tensile strength and chemical stability than Glass FRP (**GFRP**) while being significantly more economical than Carbon FRP (**CFRP**)^22,23^.

Although the flexural and shear behavior of FRP-reinforced concrete elements has been widely investigated, their application in compression members, particularly when combined with UHPC, remains underexplored. BFRP-reinforced UHPC columns are of particular interest in structural and seismic applications, where corrosion resistance and high compressive strength are crucial for long-term performance.

Recent studies have focused on improving UHPC performance through fiber hybridization, nano-material incorporation, and optimized mix designs, while others have examined the flexural and shear response of FRP-reinforced UHPC beams and slabs under static and cyclic loads. However, despite these advancements, studies investigating the cyclic response of UHPC columns reinforced with BFRP bars remain scarce.

The present study aims to bridge this research gap by conducting a detailed experimental and numerical investigation on UHPC columns reinforced with BFRP bars under cyclic lateral loading. The work evaluates the columns’ load–displacement response, failure mechanisms, and energy dissipation capacity, offering valuable insights into their potential for seismic and cyclic applications. To the best of the authors’ knowledge, this study provides one of the earliest comprehensive datasets describing the hysteretic behavior and energy dissipation characteristics of BFRP-reinforced UHPC columns, benchmarked against conventional steel-reinforced counterparts^24^.

Overall, the outcomes of this research advance current understanding of the cyclic behavior of FRP-reinforced UHPC systems and contribute to the development of reliable design recommendations and analytical models for future studies^25^. Previous studies have identified reinforced concrete columns as key structural elements governing seismic performance. Seismic hazard assessments and code-related investigations have shown that many existing reinforced concrete buildings remain vulnerable in seismic regions, primarily due to inadequate column detailing and limited ductility^26^. Field and analytical studies further confirmed that deficiencies in column design significantly increase damage concentration and collapse risk during strong ground motions^27,28^. To address these shortcomings, experimental research has explored advanced materials and alternative strengthening strategies to enhance the cyclic behavior of reinforced concrete columns. Improvements in ductility and post-cracking performance have been reported through the use of engineered cementitious composites^22^, while non-conventional reinforcement materials, such as shape memory alloys, demonstrated enhanced energy dissipation and cyclic stability under reversed loading^29^. In addition, fiber-reinforced polymer (FRP) systems have been shown to effectively improve confinement, strength, and deformation capacity of reinforced concrete columns under cyclic and seismic loading^30,31^. These studies are interpreted within the framework of established seismic design concepts, including plastic hinge behavior, ductility demand, and performance-based design principles, which are well documented in classical seismic literature^32^.

## Experimental program

The experimental work was carried out at the Reinforced Concrete Laboratory, Faculty of Engineering at Shubra, Benha University, Cairo, Egypt. A servo-controlled actuator system was used to apply the cyclic lateral loading. Six UHPC columns reinforced with Basalt Fiber Reinforced Polymer (BFRP) bars were cast and tested. All specimens were subjected to constant axial load in conjunction with cyclic lateral displacement to evaluate their structural behavior, including ultimate load-carrying capacity, strain distribution in the BFRP and steel reinforcement, crack patterns, and failure modes. Two reference columns with conventional steel reinforcement were used as a baseline for comparison with the BFRP-reinforced columns.

Further details regarding the concrete mix design, reinforcement configuration, specimen dimensions, loading protocol, and instrumentation are provided in the following sections.

### Design of specimens

**Fig. 1:**
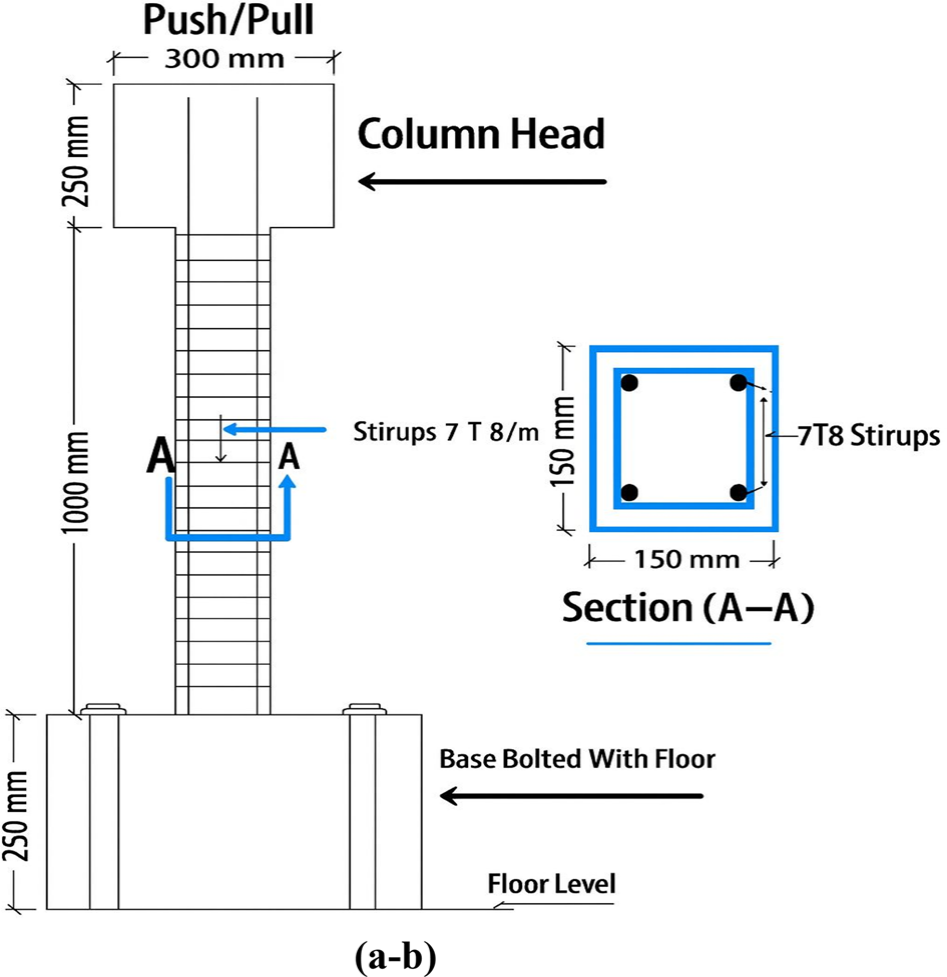
Configuration of longitudinal bars and stirrups.

**Fig. 2:**
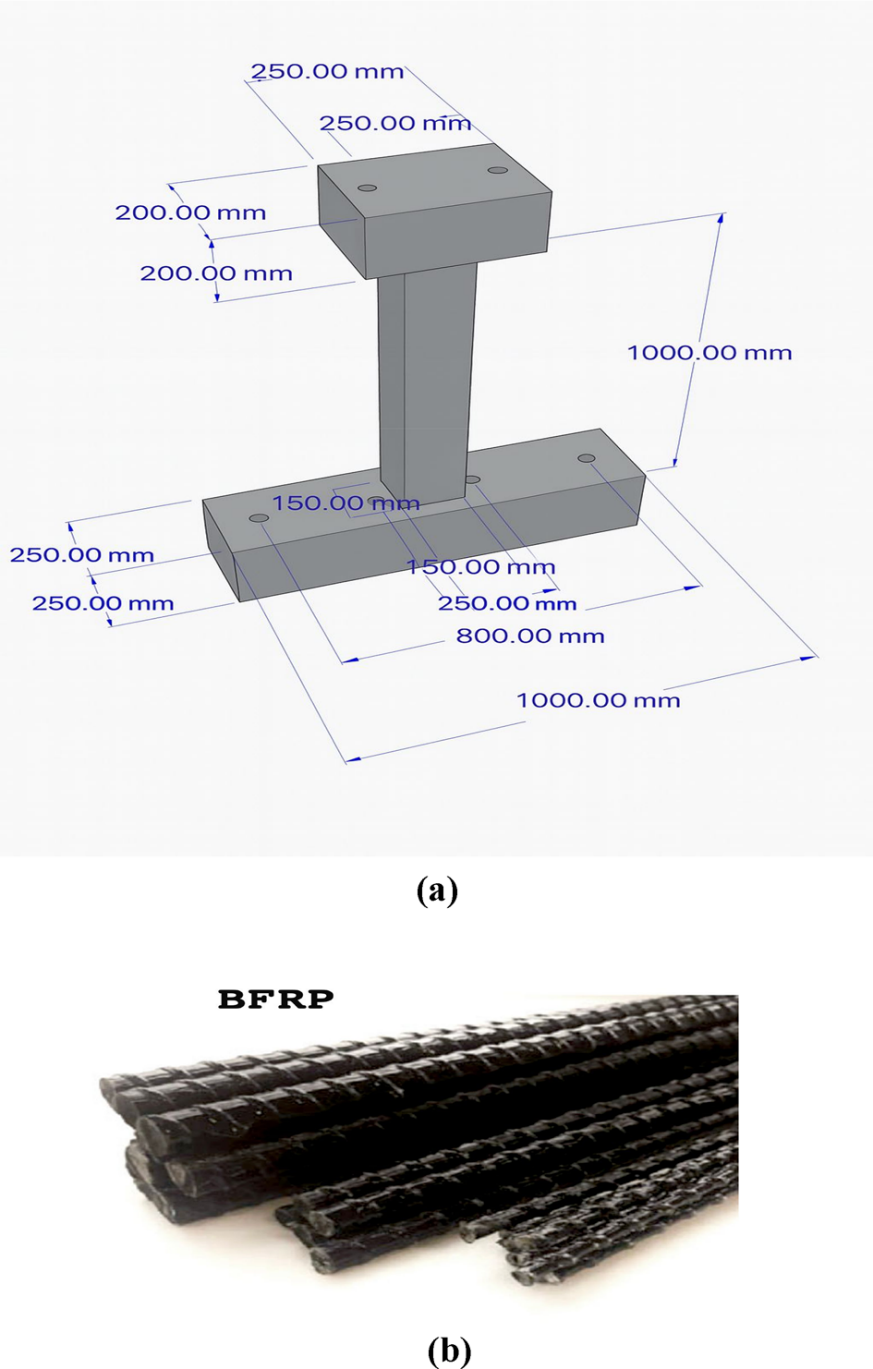
(**a**): Dimensions of the concrete column and footing, including anchor – bolt holes and prestressing ducts. (**b**). Ribbed surface geometry of the basalt fiber-reinforced polymer (BFRP) bars used in the UHPC columns, showing the helical ribs that provide strong mechanical interlock with the surrounding concrete^34^.

The experimental program consisted of two groups of reinforced concrete (RC) columns designed to investigate deformation behavior and failure modes. As shown in Fig. 1, each column had an enlarged base that was securely anchored to the laboratory’s strong floor using high-strength bolts. The top of the column was connected to a hydraulic actuator used to apply lateral cyclic loading. Each column specimen had a total height of 1500 mm and a square cross-section of 150 × 150 mm, as illustrated in Fig. 1. The footing dimensions were 1000 mm in length, 250 mm in width, and 250 mm in depth. A concrete cover of 25 mm was provided around the reinforcement^33^. The anchorage system and bolt configuration used to fix the column bases are detailed in Fig. 2(a).

### Properties of materials

#### Basalt fiber reinforced polymer (BFRP) bars

Basalt fiber reinforced polymer (BFRP) bars exhibit tensile strength approximately two to three times higher than those of conventional steel reinforcement^34^, as illustrated in Fig. 2(b). The mechanical properties of BFRP bars under tensile loading are summarized in Table 1.

**Table 1:** The mechanical properties of BFRP bars.

Property	Measured value
Specific Gravity (SG) (t/m3)	2.68
Ultimate Tensile strength (UTS) (MPa)	1100
Tensile Elastic modulus (GPa)	50
Ultimate Tensile strain (%Єu)	25

#### Concrete mix design (UHPC)

**Fig. 3:**
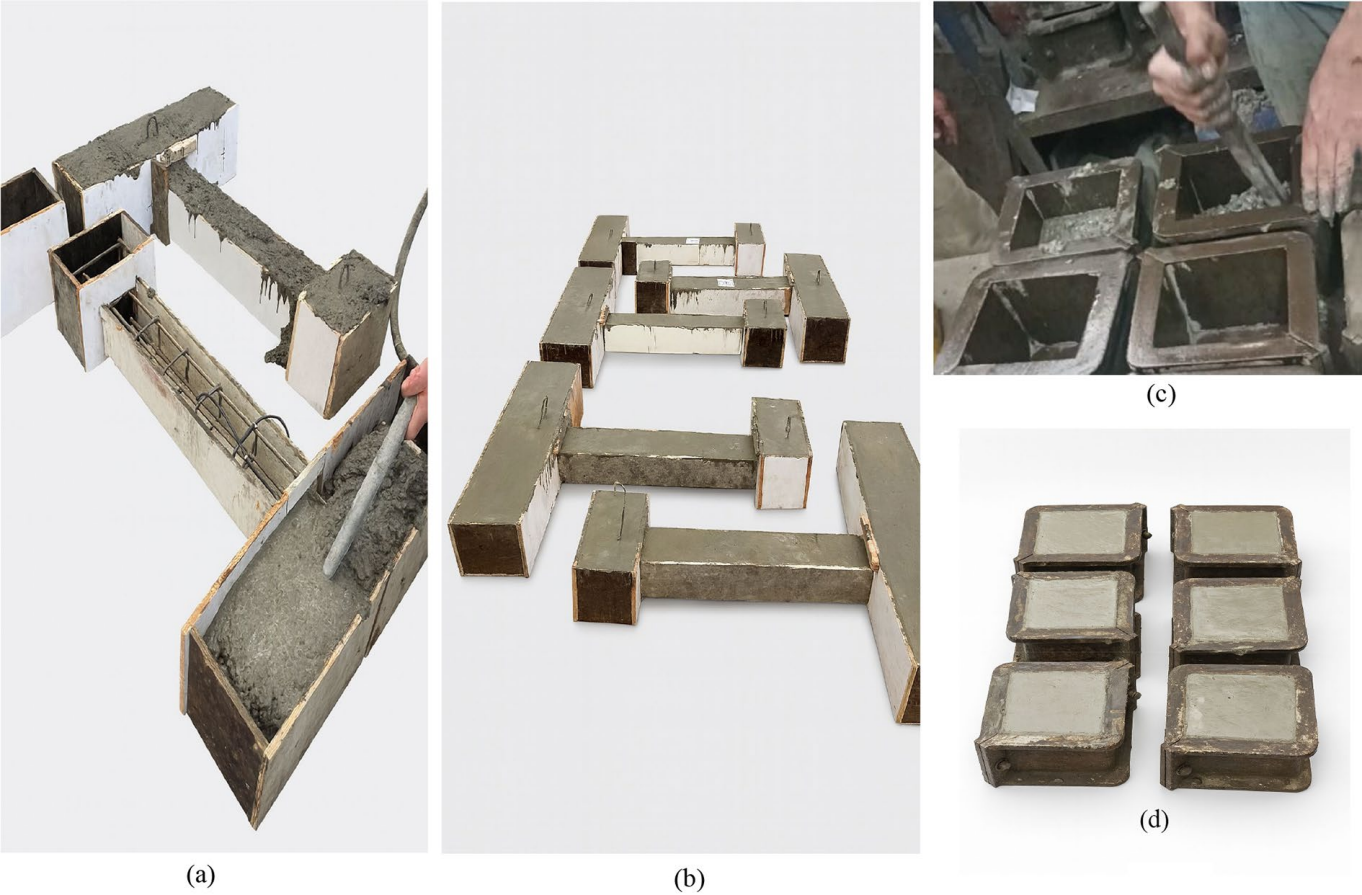
Experimental preparation stages: (**a**) Using a dynamic vibrator to ensure proper compaction and eliminate air voids; (**b**) Completed casting of ultra-high-performance concrete (UHPC) columns; (**c**) Compaction of fresh concrete in standardized test molds; (**d**) Cube specimens after casting for compressive strength testing.

The concrete used for casting the structural columns was hand-mixed in the laboratory, as shown in Fig. 3(a–c). The same mix was used for both the columns and the standard cube specimens to ensure consistency in material properties^35^. Based on several tests, the average compressive strength of the concrete ranged between 85 and 100 MPa at the time of column testing. As illustrated in Fig. 3(d), six standard cubes (150 × 150 × 150 mm) were cast from the same batch for compressive strength testing. The cube molds were filled in three equal layers, each compacted manually in accordance with the Egyptian Code of Practice (ECP 203–2020), which governs the testing of concrete and structural design procedures in Egypt^36^.

The mix proportions and quantities of materials used are summarized in Table 2. Ordinary Portland Cement (CEM I 42.5 N) was used, with basalt aggregates replacing conventional coarse aggregates (maximum size 14 mm), while fine aggregates conformed to the Egyptian Standard ES 137/2004. Additionally, silica fume was incorporated as a supplementary cementitious material to enhance the concrete’s microstructure, improve strength, and reduce permeability. The silica fume complies with ASTM C1240 specifications and exhibits high pozzolanic activity, contributing to increased durability and mechanical performance of the UHPC mix.

**Table 2:** UHPC Mix Design (kg/m^3^)^38–41^.

Materials (kg/m3)	f_cu_ = 80 MP_a_
Silica fume	85
Cement	550
Basalt aggregate	1145
Fine aggregate	430
Water	185
Super-plasticizer (Sikament R2004)	19

Sikament R2004, a chloride-free superplasticizer based on polycarboxylate ether chemistry, was used to improve the flowability and workability of the concrete mixtures without increasing their water content^37^. This admixture effectively disperses cement particles, reducing the water-to-cement ratio and enhancing the concrete’s mechanical strength and durability. Moreover, Sikament R2004 promotes better particle packing by improving the homogeneity of the concrete matrix and reducing voids. It also extends slump retention time, facilitating easier handling and placement of the concrete, which is especially important for low water-to-cement ratio mixtures such as UHPC. Compatible with ordinary Portland cement and supplementary cementitious materials like silica fume, its chloride-free composition prevents corrosion of steel or fiber-reinforced polymer reinforcements.

The selected proportions of silica fume and superplasticizer played a decisive role in achieving a highly workable UHPC mix without segregation, while maintaining excellent cohesion. The silica fume enhanced particle packing and reduced internal voids, leading to a denser and less permeable matrix^42^. Meanwhile, the polycarboxylate-based superplasticizer improved flowability and particle dispersion at the low w/c ratio of 0.20, ensuring uniform compaction and homogeneity throughout the mix. This synergistic interaction directly contributed to the high compressive strength values achieved (85–100 MPa) and the refined microstructure of the UHPC columns, consistent with previously reported findings by **Aïtcin**^43^ and **Graybeal**^44^.

These proportions were optimized through preliminary trial mixes to achieve the desired balance between workability and mechanical performance, ensuring consistency among all tested specimens.

#### Results of the compressive strength test

Compressive strength tests were performed on standard concrete cube specimens with dimensions of 150 × 150 × 150 mm after curing periods of 7 and 28 days. The tests were conducted using a universal testing machine with a maximum load capacity of 2000 kN, following the procedures specified in the Egyptian Code of Practice (ECP 203–2020). The compressive strength values presented in Table 3 represent the average results of three specimens tested at each curing age. The results confirmed the uniformity and reliability of the UHPC mix design used for casting the column specimens.

**Table 3:** Compressive strength tests results.

Cubes	Compressive Strength (MPa)	
	7 days	28 days
C1	60.75	79.9
C2	75.7	80.2
C3	78.5	82.7
Avg	71.65	80.93

#### Experimental setup and loading protocol

**Fig. 4:**
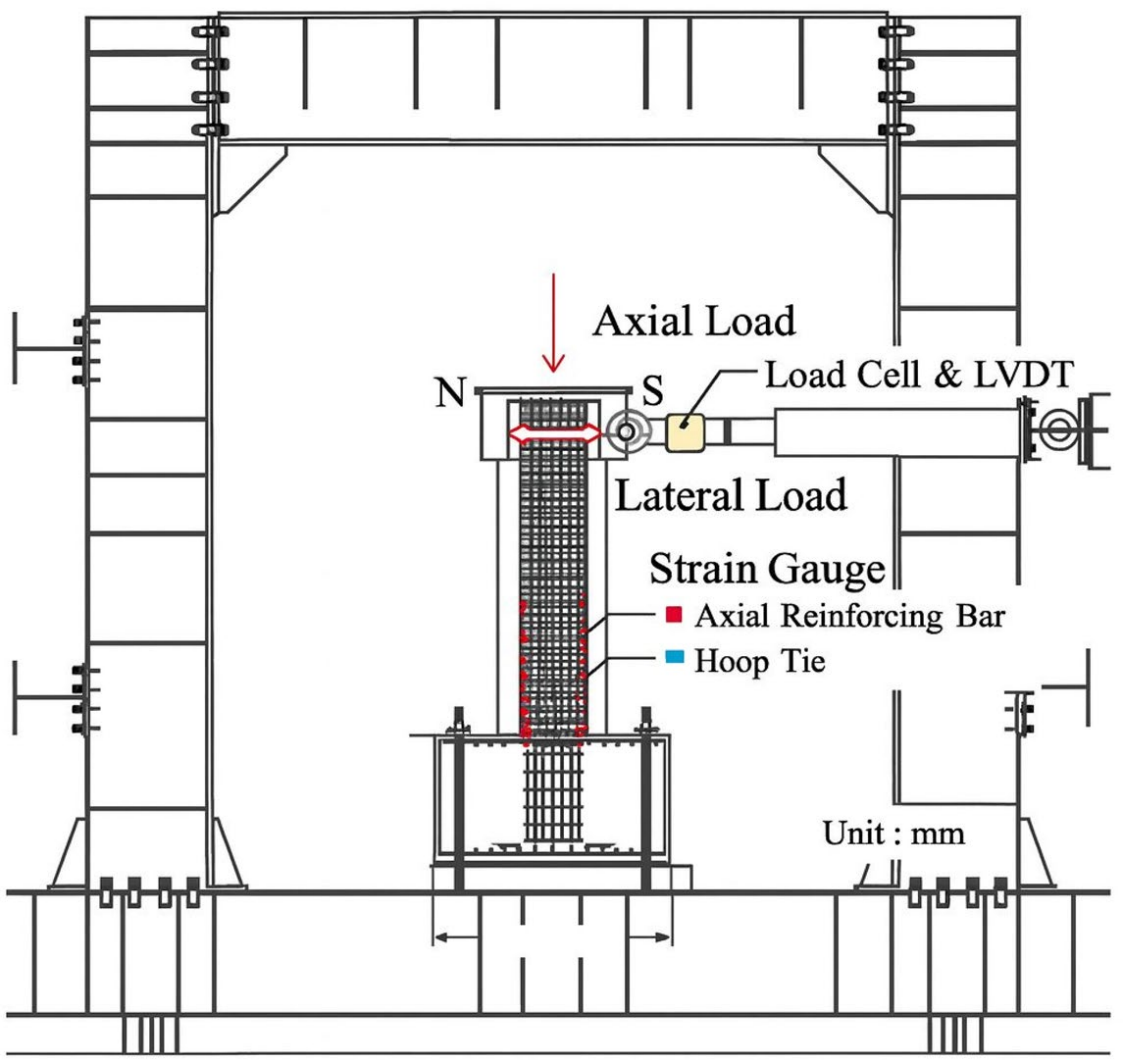
Experimental setup for lateral cyclic loading of RC column^46^.

A comprehensive instrumentation system was employed to continuously record and monitor all experimental data during testing. The setup integrated strain gauges, Linear Variable Differential Transformers (LVDTs), and load cells to capture accurate measurements of strain, displacement, and applied loads, respectively. Strain gauges were attached to both the longitudinal reinforcement bars and the concrete surface to measure localized deformations, while LVDTs were positioned along the column height to track lateral displacements under cyclic loading^45^. Load cells were installed to monitor both axial and lateral forces throughout the test. All sensors were connected to a centralized data acquisition system, which automatically recorded measurements in real time using specialized computer software for visualization and storage. This configuration ensured high precision and reliability of the collected data, which was critical for evaluating the mechanical behavior, failure mechanisms, and performance of the BFRP-reinforced UHPC columns. The overall experimental setup for cyclic lateral loading is shown in Fig. 4. A summary of the specimen grouping and reinforcement configurations used in this experimental program is provided in Table 4.

**Table 4:** Specimen’s description.

Groups	Column ID	RFT. Type	Long. RFT	Trans. RFT
Group A	C1‑A	Steel	4 Ø 10	7Ø8/m
	C2‑A	Steel	4 Ø 10	7Ø8/m
Group B	C1‑B	Basalt	4 Ø 10	7Ø8/m
	C2‑B	Basalt	4 Ø 10	7Ø8/m
	C3‑B	Basalt	4 Ø 12	7Ø8/m
	C4‑B	Basalt	4 Ø 12	7Ø8/m

**Fig. 5:**
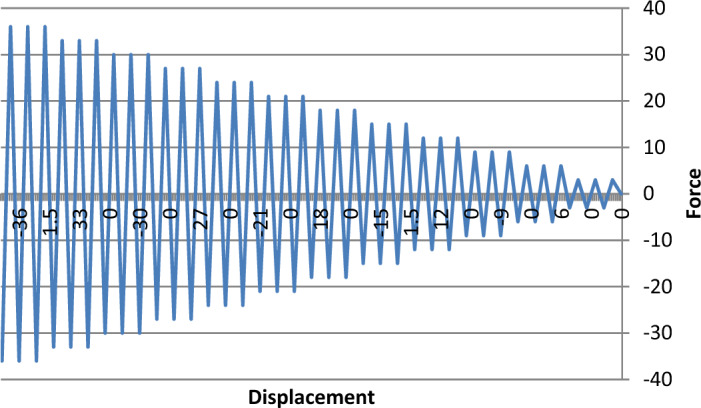
Cyclic loading protocol applied in this study.

The actuator was connected to the column head through a steel loading arm, and displacement-controlled loading cycles were applied following a predefined reversed cyclic protocol^47^. The loading pattern followed a quasi-static sequence with gradually increasing drift levels until failure^48^. The drift ratio at each cycle was controlled to capture stiffness degradation, energy dissipation, and strength deterioration, as shown in Fig. 5.

To simulate seismic loading conditions, a displacement-controlled reversed cyclic loading protocol was adopted, as illustrated in Fig. 5. The sequence consisted of three fully reversed cycles at each displacement amplitude level. The initial lateral displacement was set to ± 3 mm, and after each set of three cycles, the amplitude was increased in 3 mm increments. This staged loading continued until either the specimen experienced significant degradation or reached the maximum amplitude of ± 36 mm.

Throughout the entire test, key parameters including lateral load, lateral displacement, strain in reinforcement, and crack development were continuously monitored. Four LVDTs were installed at heights ranging between 50 and 100 mm above the column base on both sides to measure lateral deformations, in addition to one LVDT mounted at the top of the column to record total lateral displacement, as shown in Fig. 4.

This integrated instrumentation and loading setup provided a comprehensive framework for evaluating the columns’ hysteretic behavior, including stiffness degradation, energy dissipation, and failure progression, thereby offering valuable insight into their seismic performance under cyclic lateral loading^48^. To ensure the accuracy of displacement measurements, the LVDT installed at the column top was rigidly connected to the actuator and column cap, thereby recording the total lateral displacement without any influence from base movement. The additional LVDTs distributed along the column height were used to capture the deformation shape and verify that any base rotation or slippage was negligible. This was confirmed experimentally, as the column footing was firmly anchored to the laboratory strong floor, preventing any measurable movement at the interface.

## Experimental results

### Ultimate load and deformation behavior

**Fig. 6:**
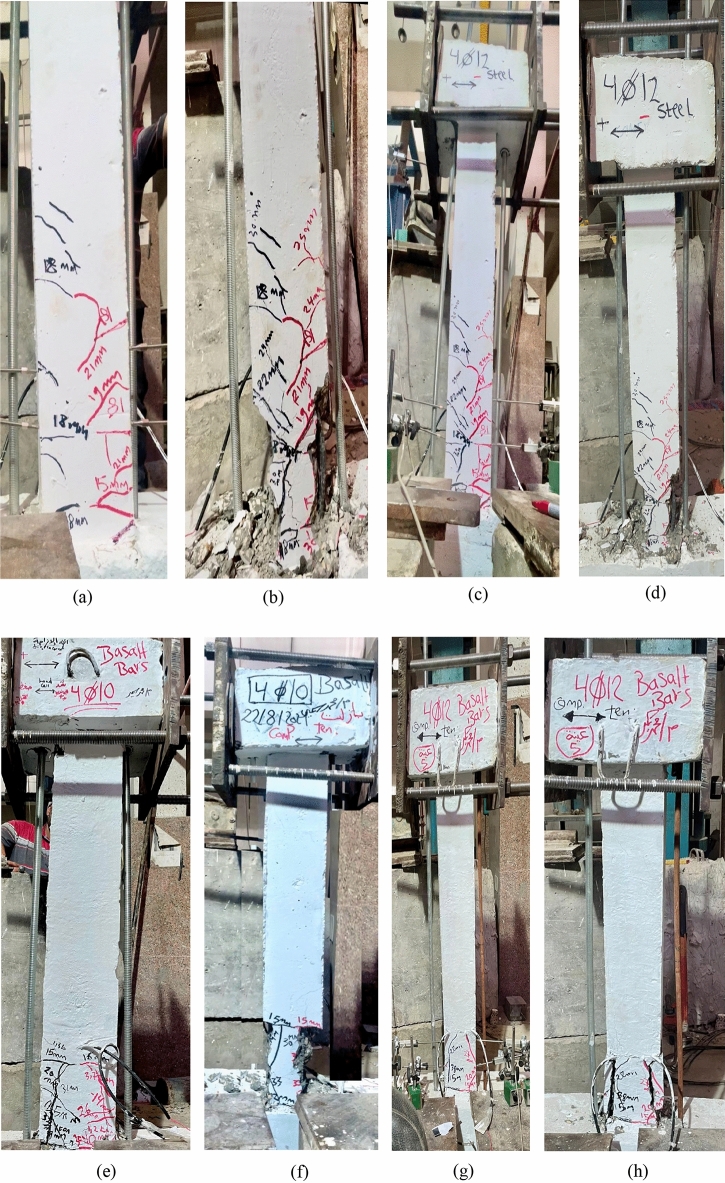

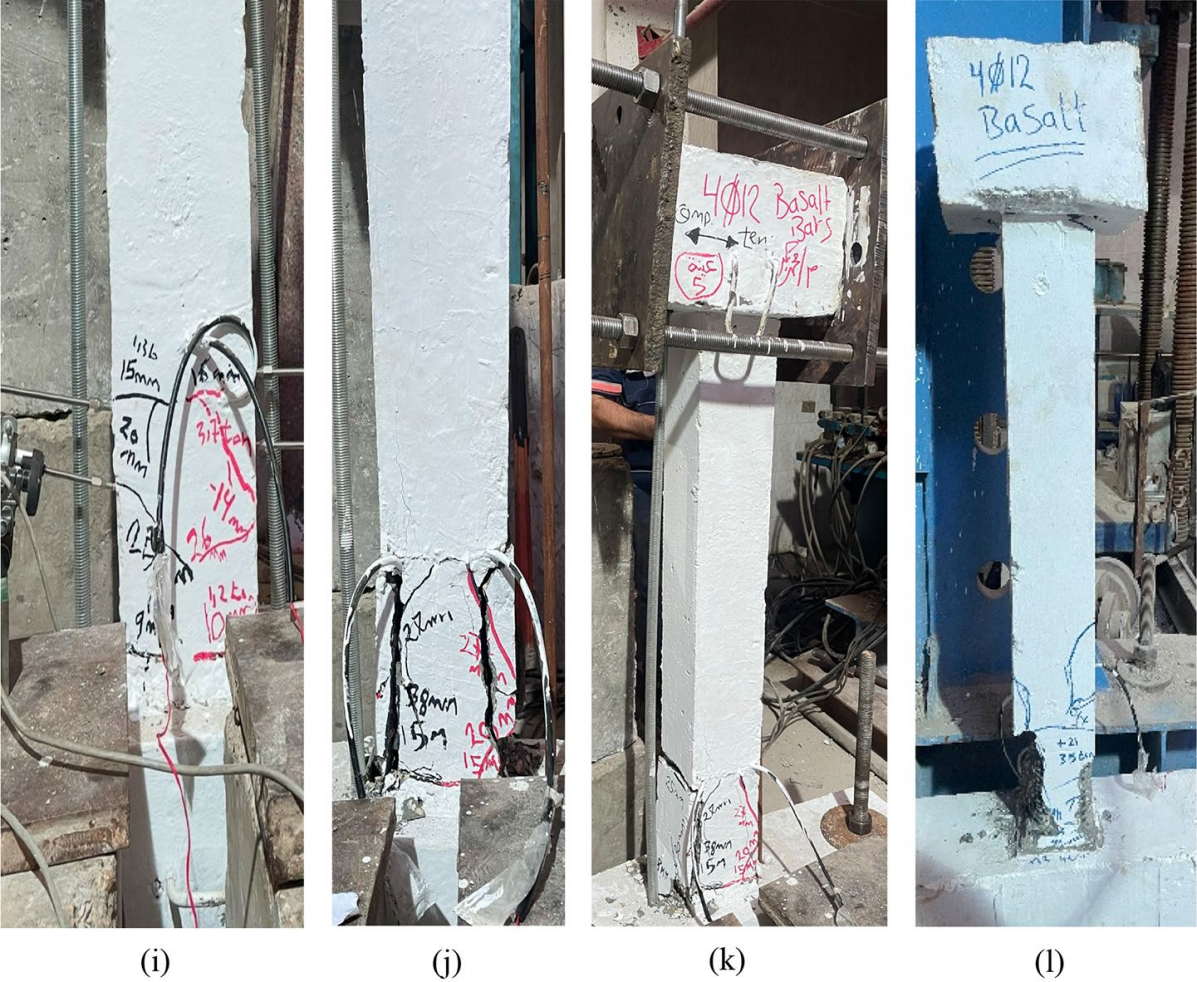
Damage progression in UHPC column specimens under cyclic lateral loading: Steel-reinforced specimens: (**a–d**) Flexural crack initiation followed by concrete spalling and final failure in specimens reinforced with 4Ø10 mm and 4Ø12 mm steel bars. Basalt-reinforced specimens (4Ø10 mm): (**e–h**) Flexural crack initiation and subsequent concrete spalling leading to final failure in two specimens reinforced with 4Ø10 mm basalt bars. Basalt-reinforced specimens (4Ø12 mm): (**i–l**) Flexural crack initiation and concrete spalling leading to final failure in two specimens reinforced with 4Ø12 mm basalt bars.

This section presents the experimental results regarding the ultimate lateral load capacity, deformation characteristics, and failure mechanisms of the tested UHPC columns under cyclic lateral loading. The observed damage patterns and crack propagation are shown in Fig. 6 (a–l), and a summary of the key results is listed in Table 5.

**Table 5:** Test results.

Column ID	Ultimate load (kN)	Def. at Ult. load (mm)	Std. Dev. (Ultimate load) kN	Std. Dev. (Deflection) mm
C1-A	10.85	36.747	—	—
C2-A	14.52	35.879	—	—
C3-B	14.26	35.773	0.523	0.176
C4-B	15	35.524	0.523	0.176
C5-B	18.51	36.565	0.813	0.251
C6-B	19.66	36.92	0.813	0.251

**Two reference specimens reinforced with conventional steel were tested:**

- Specimen C1-A was reinforced with 4Φ10 mm longitudinal steel bars and 7Φ8/m steel stirrups.
- Specimen C2-A included 4Φ12 mm longitudinal steel bars and the same stirrup configuration.

These control specimens were used to benchmark the performance of similar columns reinforced with basalt fiber-reinforced polymer (BFRP) bars.

**The BFRP-reinforced specimens were as follows:**

- C3-B and C4-B used 4Φ10 mm longitudinal basalt bars and BFRP stirrups.
- C5-B and C6-B used 4Φ12 mm longitudinal basalt bars and BFRP stirrups.

All specimens experienced typical flexural failure modes initiated by the formation of fine cracks, which gradually propagated under increasing displacement cycles. In the steel-reinforced specimens, cracks widened significantly with increasing lateral drift, eventually leading to reinforcement yielding, concrete cover spalling, and failure in the plastic hinge region due to crushing and bar buckling.

In contrast, the BFRP-reinforced columns (C3-B to C6-B) exhibited superior performance. They sustained higher ultimate lateral loads and showed significantly less cracking throughout the test. For instance, specimen C3-B reached a peak lateral load of 14.26 kN, while C4-B achieved 15.00 kN, representing a strength increase of approximately 23.91% compared to C1-A. Similarly, C5-B and C6-B recorded peak loads of 18.51 kN and 19.66 kN, respectively, reflecting a load capacity increase of about 26.14% over C2-A.

The improved performance of the BFRP-reinforced specimens is attributed to the high tensile strength and corrosion resistance of basalt bars. Additionally, the crack widths remained significantly narrower than in steel-reinforced columns, indicating better post-cracking stiffness and enhanced confinement efficiency of BFRP stirrups.

These findings align well with the general behavior reported in the literature for FRP-reinforced concrete columns under cyclic or seismic loading. Previous studies on GFRP and CFRP-reinforced columns have also shown improved load-carrying capacity, reduced crack widths, and stable hysteretic responses compared with steel-reinforced counterparts. However, the combination of BFRP reinforcement with UHPC in the present study led to even greater strength enhancement and deformation capacity. This synergy arises from the dense UHPC matrix, which improves the bond and stress transfer along the ribbed surface of the BFRP bars, thereby minimizing slippage and enhancing the overall confinement effect. Such behavior supports the suitability of BFRP-UHPC systems for seismic-resistant structural applications.

Although BFRP bars are non-ductile and possess a lower elastic modulus than steel, their superior tensile strength delayed rupture of the longitudinal reinforcement. The enhanced confinement observed in BFRP-reinforced columns primarily originated from the BFRP stirrups, which despite their lower modulus provided effective passive confinement to the concrete core once the cover spalled. Because BFRP does not yield, this confining pressure was maintained at higher strain levels, preventing sudden concrete crushing and allowing the specimens to sustain greater deformation before failure. This confinement mechanism is clearly reflected in the load–displacement curves, where BFRP-reinforced specimens exhibited more stable hysteretic behavior and slower stiffness degradation compared to their steel-reinforced counterparts^49^.

The superior performance and effective crack control observed in the BFRP-reinforced specimens are also attributed to the strong mechanical interlock developed by the ribbed (helically profiled) surface of the basalt bars with the surrounding UHPC matrix, as illustrated in Fig. 2(b). Previous studies^50,51^ have shown that rib geometry significantly increases bond strength and restricts bar–concrete slip, thereby enhancing stress-transfer efficiency and delaying crack initiation and widening under cyclic loading.

These results are consistent with reported bond–slip behavior of ribbed BFRP bars in UHPC and normal concretes, where ribbing or helical profiling outperforms smooth and sand-coated surfaces in bond capacity and durability. This observation, together with the ribbed geometry shown in Fig. 2(b), explains the enhanced cyclic performance demonstrated in the current study.

### Cracking patterns of concrete columns

The cracking behavior of the tested concrete columns under cyclic lateral loading was systematically observed and documented, as illustrated in Fig. 6(a–l). All specimens exhibited characteristic flexural damage mechanisms, particularly in the plastic hinge zone near the base of the columns. The crack patterns evolved progressively with increasing drift levels and cyclic degradation.

### Lateral load–displacement hysteresis loops

The lateral load–displacement response of the tested specimens under reversed cyclic loading exhibited typical hysteretic behavior. The lateral displacement was expressed in terms of the drift ratio, calculated by dividing the lateral displacement recorded at the top of the column (i.e., actuator displacement) by the effective height of the column (1125 mm), measured from the top of the footing to the mid-height of the column cap.

**Fig. 7:**
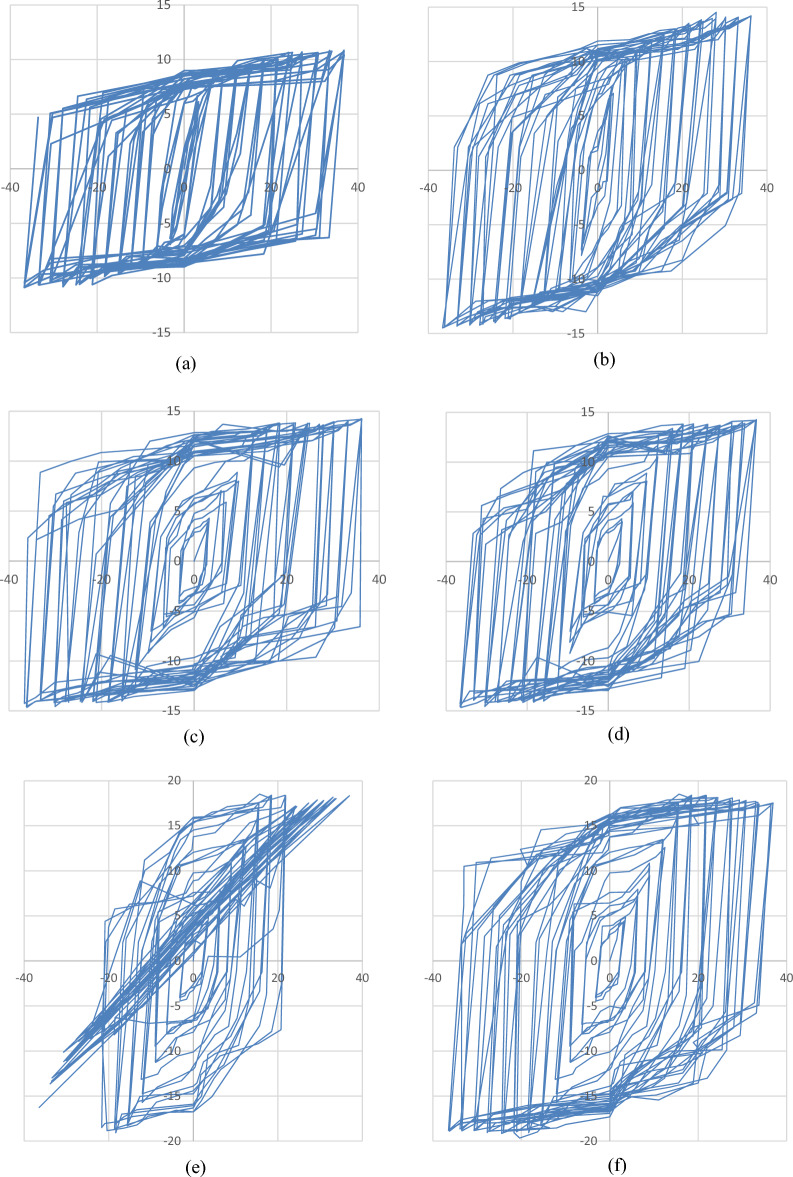
Lateral load–displacement hysteresis loops of UHPC column specimens: (**a–b**) Steel-reinforced specimens with 4Ø10 mm and 4Ø12 mm bars; (**c–d**) Basalt-reinforced specimens with 4Ø10 mm bars; (**e–f**) Basalt-reinforced specimens with 4Ø12 mm bars.

Each complete loading cycle generated a hysteresis loop, illustrating the relationship between lateral load and displacement. The hysteretic responses of the tested columns are presented in Fig. 7(a–f), corresponding to columns reinforced with 4Ø10 mm and 4Ø12 mm steel bars, as well as those reinforced with basalt bars of the same diameters, respectively.

All plots share a unified scale of lateral displacement (mm) on the x-axis and lateral load (kN) on the y-axis to enable direct visual comparison of the hysteretic behavior among specimens.

### Lateral load–displacement behavior

**Fig. 8:**
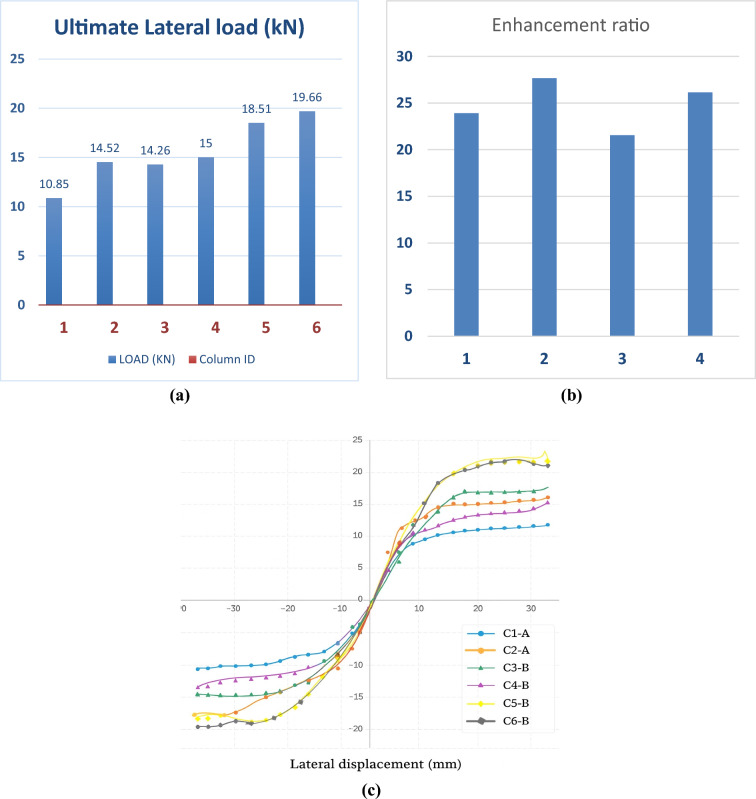
Comparison of lateral load capacities and enhancement ratios of UHPC column specimens: (**a–b**) Peak lateral load capacities (kN) for different reinforcement configurations and the corresponding percentage enhancement relative to the steel-reinforced control specimens. (**c**) Envelope curves of lateral load–displacement responses for all tested specimens with various reinforcement configurations and axial load ratios.

The experimental findings revealed a notable improvement in the lateral load-bearing capacity of columns reinforced with basalt bars. This enhancement was particularly evident when basalt longitudinal reinforcement was utilized, contributing to better confinement and structural integrity. The increase in ultimate lateral resistance exceeded 25% in comparison to conventional steel-reinforced specimens, as detailed in Table 5 and illustrated in Fig. 8(a–b). These results highlight the effectiveness of basalt reinforcement in improving seismic performance under cyclic loading conditions.

### Stiffness degradation and energy dissipation

This subsection quantifies the cyclic response in terms of (i) stiffness degradation versus drift ratio and (ii) cumulative energy dissipation. The analysis was performed for all six specimens using the measured load–displacement histories.

**Fig. 9:**
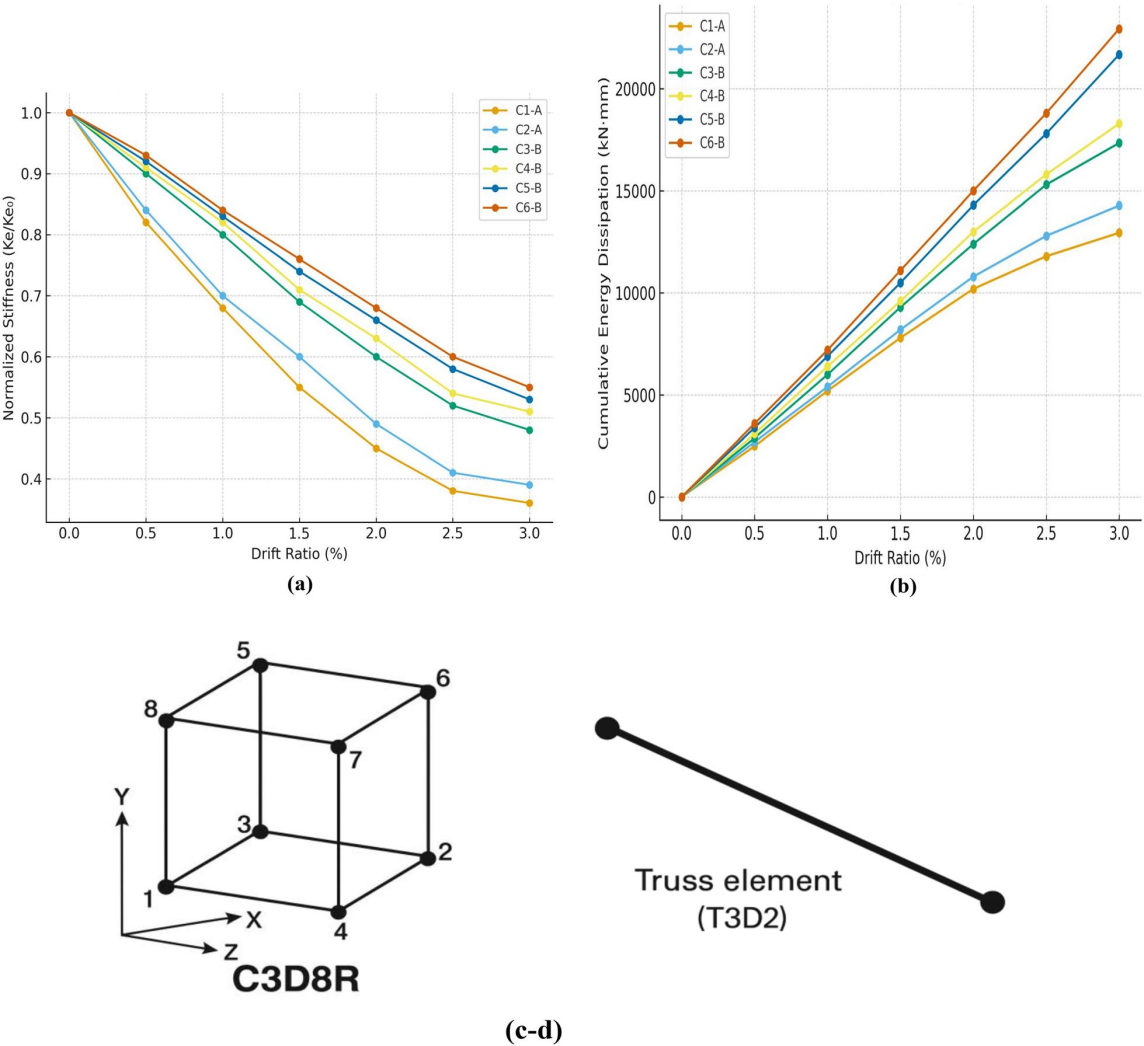
(**a**) Stiffness Degradation vs Drift Ratio. (**b**) Cumulative Energy Dissipation vs Drift Ratio. (**c-d**) T3D2 truss and C3D8R solid elements used for modeling reinforcement and concrete, respectively.

As shown in Fig. 9(a), all specimens exhibited progressive stiffness loss with increasing drift. The steel-reinforced controls (C1-A, C2-A) showed a sharper post-yield decay, whereas the BFRP-reinforced columns (C3-B to C6-B) maintained higher residual stiffness at comparable drifts, consistent with their narrower crack widths and reduced damage accumulation.

The stiffness degradation curves (Fig. 9-a) were constructed by calculating the secant stiffness at each load cycle as Ke = P/Δ, normalized by the initial-cycle stiffness Ke0. The normalization allows for direct comparison between specimens of different reinforcement types and bar diameters.

Figure 9(a). Stiffness degradation versus drift ratio for all specimens. The secant stiffness Ke at each cycle was computed as Ke = P/Δ and normalized by the initial-cycle value Ke0. BFRP-reinforced columns retained higher normalized stiffness at larger drift ratios relative to steel-reinforced controls.

The cumulative energy dissipation increased monotonically with drift for all specimens (Fig. 9-b). Notably, the BFRP-reinforced columns showed sustained energy accumulation at higher drift levels, indicating stable hysteretic behavior and delayed damage localization compared with the steel-reinforced controls.

Energy dissipation was computed by integrating the area enclosed within each hysteresis loop and accumulating it across loading cycles. The results confirm that the UHPC–BFRP system exhibited superior energy absorption and more stable cyclic resistance, owing to the composite’s enhanced crack control and the non-corroding nature of basalt reinforcement.

Figure 9(a-b). Cumulative energy dissipation versus drift ratio obtained by integrating the enclosed area of each hysteresis loop and accumulating over cycles. BFRP-reinforced columns exhibit stable and gradually increasing energy absorption with drift.

The results summarized in Table 6 and illustrated in Figs. 9(a)–9(b) demonstrate that the BFRP-reinforced UHPC columns exhibited up to 40–50% higher cumulative energy absorption and retained 25–35% greater normalized stiffness at comparable drift ratios compared with steel-reinforced counterparts.

**Table 6:** Summary of stiffness retention and cumulative energy dissipation for each specimen.

Specimen	Reinforcement	Bar Diameter (mm)	Max Drift (%)	Final Ke/Ke₀	Cumulative Energy (kN·mm)
C1-A	Steel	10	2.80	0.36	12,950
C2-A	Steel	12	3.05	0.39	14,280
C3-B	BFRP	10	2.85	0.48	17,340
C4-B	BFRP	10	3.00	0.51	18,290
C5-B	BFRP	12	2.90	0.53	21,680
C6-B	BFRP	12	3.10	0.55	22,940

In addition to stiffness and energy dissipation, the ductility performance of the tested columns was evaluated. Table 7 presents the calculated ductility indices and energy dissipation ratios for all specimens. (see Table 7).

**Table 7:** Summary of ductility index and energy dissipation ratio for tested UHPC columns.

Specimen ID	Reinforcement Type	Bar Diameter (mm)	Ultimate Drift (%)	Yield Drift (%)	Ductility Index (μ = Δu/Δy)	Energy Dissipation Ratio (EBFRP/E Steel)
C1-A	Steel	10	2.80	0.85	3.29	–
C2-A	Steel	12	3.05	0.90	3.39	–
C3-B	BFRP	10	2.85	1.20	2.38	1.34
C4-B	BFRP	10	3.00	1.25	2.40	1.28
C5-B	BFRP	12	2.90	1.30	2.23	1.52
C6-B	BFRP	12	3.10	1.35	2.29	1.46

## Finite element modelling and sensitivity analysis

A nonlinear finite element analysis (FEA) was conducted using ABAQUS to simulate the behavior of ultra-high-performance concrete (UHPC) columns reinforced with basalt fiber-reinforced polymer (BFRP) bars under cyclic lateral loading. The material properties of UHPC, steel reinforcement, and BFRP bars were defined based on both experimental data and ABAQUS technical documentation^52^.

**Fig. 10:**
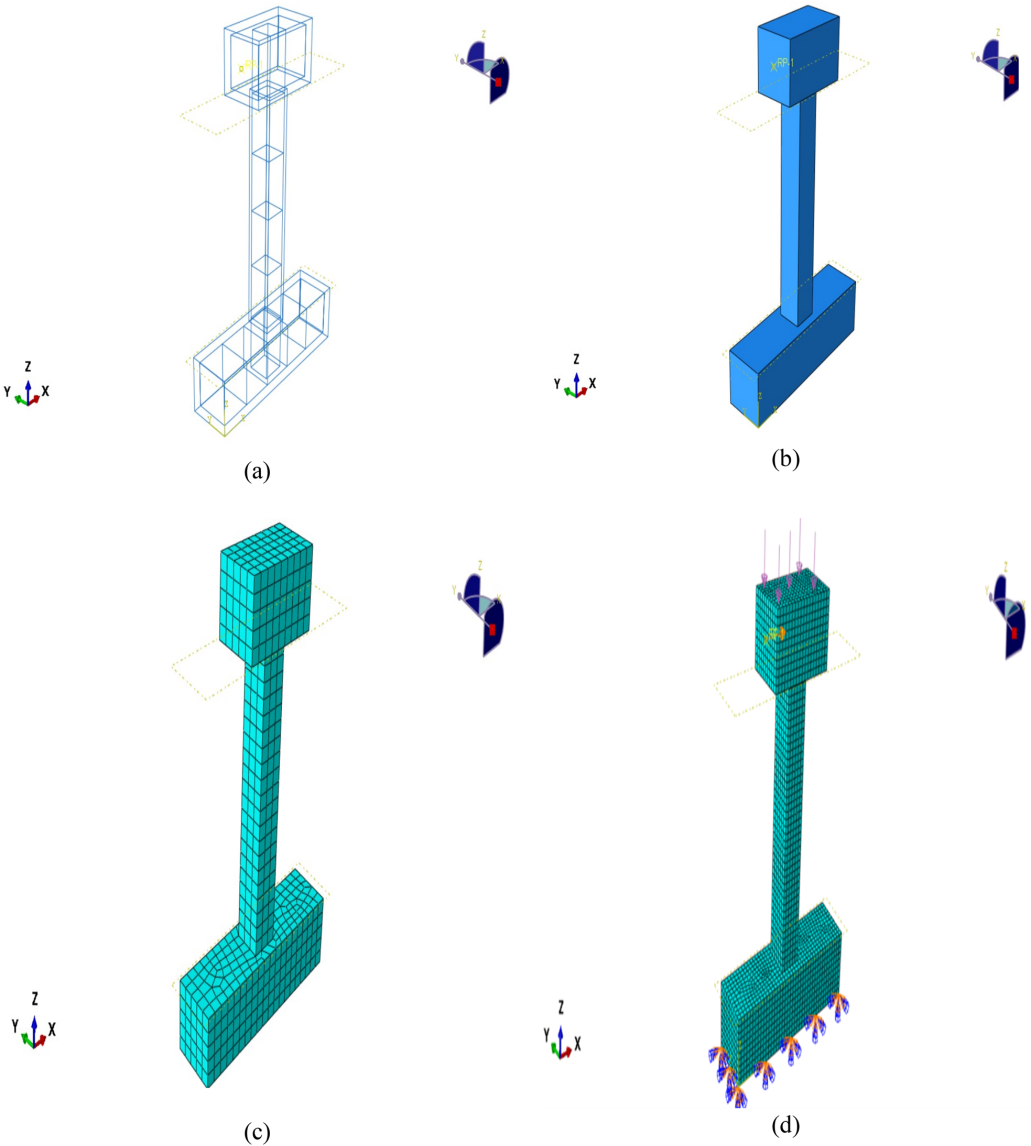
Reinforcement details, concrete specimens, and typical finite element meshing: (**a**) Reinforcement of specimens; (**b**) Concrete of specimens; (**c–d**) Typical finite element meshing.

The numerical model was validated by comparing the simulated load–displacement responses with the experimental results, demonstrating strong agreement and confirming the model’s accuracy and reliability for subsequent parametric studies^53^. In the simulation, three-dimensional eight-node reduced integration solid elements (C3D8R) were used to model the concrete, while two-node truss elements (T3D2) were employed for both longitudinal and transverse reinforcements, including steel and BFRP bars, as illustrated in Fig. 10(a–d). The selection of these element types was based on their established ability to accurately represent the nonlinear behavior of reinforced concrete structures under cyclic loading.

A detailed sensitivity analysis was also carried out to verify the numerical robustness of the developed model. A mesh convergence study was performed by progressively refining the element size of the concrete domain until the change in ultimate lateral load capacity was less than 2%. The analysis confirmed that the chosen C3D8R and T3D2 elements provided mesh-independent and numerically stable results. Moreover, the parameters of the Concrete Damage Plasticity (CDP) model—such as dilation angle, eccentricity, and the ratio of biaxial to uniaxial compressive strength—were carefully calibrated using the experimentally obtained stress–strain relationships for UHPC. This calibration ensured realistic simulation of stiffness degradation and post-peak behavior under cyclic loading. The validated FE model showed stable convergence and excellent agreement with experimental hysteresis curves, confirming its robustness and reliability for subsequent parametric studies (see Fig. 9(c-d)).

### Reinforced concrete columns modelling

Three-dimensional finite element modeling was performed using ABAQUS to analyze the behavior of UHPC columns reinforced with BFRP bars under cyclic loading. The concrete was modeled using solid elements, while the longitudinal and transverse reinforcements were represented by truss elements. Appropriate boundary conditions and loading protocols were applied to replicate the experimental setup. Material properties were defined based on laboratory results to ensure the accuracy of the simulation.

### Ultimate load-carrying capacity from nonlinear finite element analysis

Table 8 presents the ultimate load-carrying capacities of all simulated columns. The first reference column, C1-A, was reinforced with 4Φ10 mm longitudinal steel bars and transverse stirrups spaced at 7Φ8/m and failed at an ultimate load of 11.22 kN. The second reference specimen, C2-A, had an upgraded reinforcement configuration using 4Φ12 mm vertical steel bars and the same transverse reinforcement arrangement (7Φ8 mm/m), and exhibited an increased load capacity of 14.20 kN. Columns C3-B to C6-B, which were reinforced with BFRP bars, showed further enhancement in performance, with ultimate failure loads ranging from 14.40 kN to 20.49 kN. An analytical improvement in load capacity was observed in these columns, with increases of up to 22.08% and30.69%, respectively, compared to the steel-reinforced control specimen^54,55^.

**Table 8:** Comparison between experimental and numerical results.

**Experimental**			**Numerical**			
Column No	P Exp	Δ Exp	PNum	ΔNum	Pf Exp./ Pu Num	Δf Exp./ Δ u Num
	**kN**	mm	**kN**	mm		
C1-A	10.85	36.74	11.22	36.99	0.96	0.99
C2-A	14.52	35.87	14.20	34.55	1.02	1.03
C3-B	14.26	35.77	14.40	35.00	0.99	1.02
C4-B	15.00	35.52	14.80	36.00	1.01	0.98
C5-B C6-B	18.51 19.66	36.56 36.92	18.36 20.49	35.50 35.66	1.00 0.95	1.03 1.03
Average	-	-	-	-	0.98	0.99
Standard dev	-	-	-	-	0.01	0.03

### Parametric study and failure modes of specimen and damage analysis

The analyzed columns exhibited progressive concrete damage that developed gradually with the increase in the number of applied cyclic loading cycles. In the numerical simulation, concrete damage was quantified using the DAMAGET index in Abaqus, as described in^56^, where higher DAMAGET values indicate more severe deterioration.

**Fig. 11:**
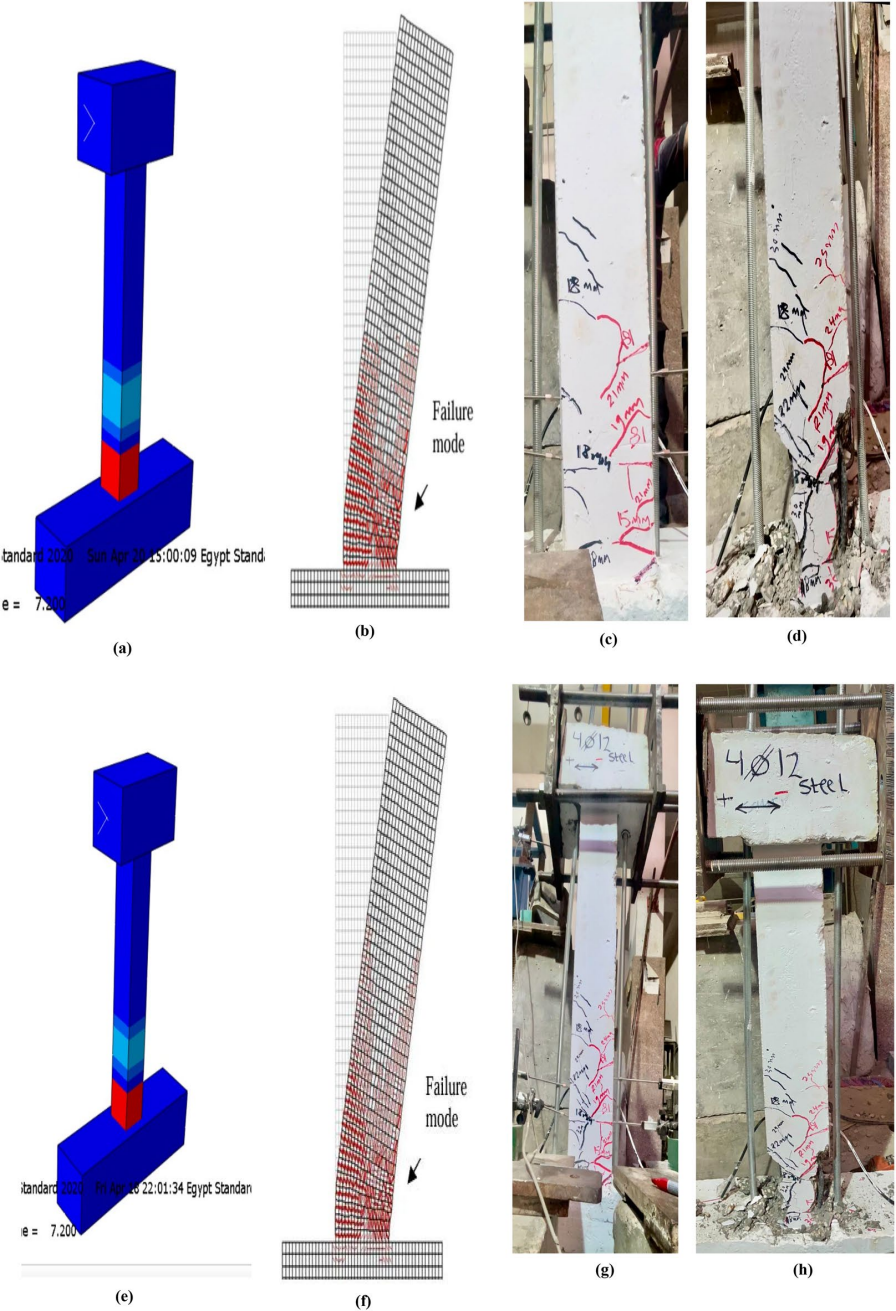

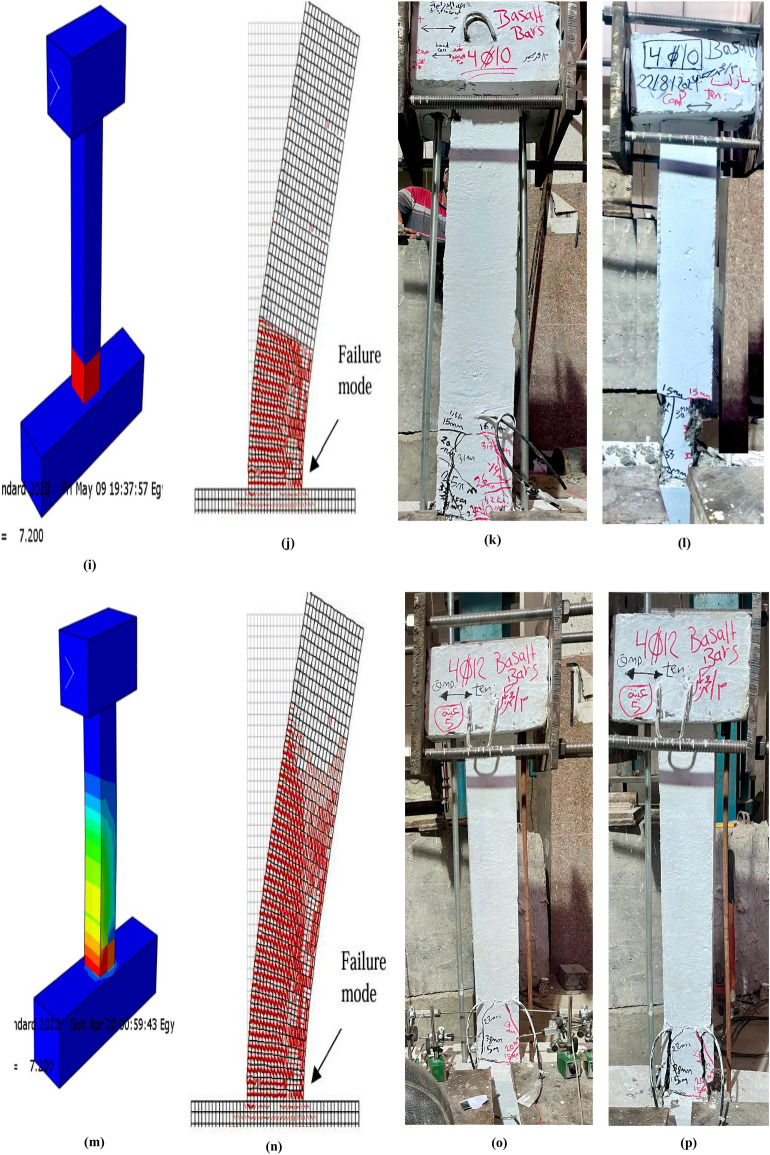
Final deformation patterns, stress distribution, and failure modes of UHPC column specimens under cyclic lateral loading: Specimen C1-A (10 mm steel bars) (**a**) Concrete stress pattern during cyclic loading; (**b**) FE-predicted DAMAGET contour; (**c**) Experimental crack pattern; (**d**) Experimental failure mode. The close similarity between the experimental and numerical damage propagation confirms the validity of the FE model in replicating real structural behavior^57^. Specimen C2-A (12 mm steel bars) (**e**) Concrete stress distribution during cyclic loading; (**f**) FE-predicted DAMAGET contour; (**g**) Experimentally observed crack pattern; (**h**) Experimental failure mode. Specimen C3-B (10 mm basalt bars) (**i**) Concrete stress distribution during cyclic loading; (**j**) FE-predicted DAMAGET contour; (**k**) Experimentally observed crack pattern; (**l**) Experimental failure mode. Specimen C4-B (12 mm basalt bars) (**m**) Concrete stress distribution during cyclic loading; (**n**) FE-predicted DAMAGET contour; (**o**) Experimental crack pattern; (**p**) Experimental failure mode.

Figure 11 illustrates the evolution of concrete damage at different loading stages for the tested specimens Fig. 11(a-p). During the initial cracking phase, minor damage appeared near the base of the columns. As the intensity and number of cyclic loads increased, the damaged area extended progressively upwards. At the ultimate and failure stages, high DAMAGET values were observed up to nearly the mid-height of the columns, indicating significant damage propagation^57^.

Figure 11 presents a direct comparison between the experimental and numerical results for representative specimens (C1-A to C4-B). It shows the concrete stress distribution, FE-predicted DAMAGET contours, and the experimentally observed crack patterns and failure modes for both steel- and BFRP-reinforced columns. This layout enables a clear visual assessment of the FE model’s accuracy in reproducing actual damage propagation, crack inclination, and failure localization. The close agreement between the experimental and numerical results demonstrates the model’s capability to realistically simulate stiffness degradation and progressive concrete crushing under cyclic lateral loading.

A simplified parametric study was also conducted using the validated finite element model to investigate the influence of cyclic loading amplitude and concrete compressive strength on the structural response of the columns under lateral loading conditions.

The strong agreement between experimentally observed and numerically predicted damage evolution demonstrates the accuracy and reliability of the finite element model in capturing the actual structural response.

**Fig. 12:**
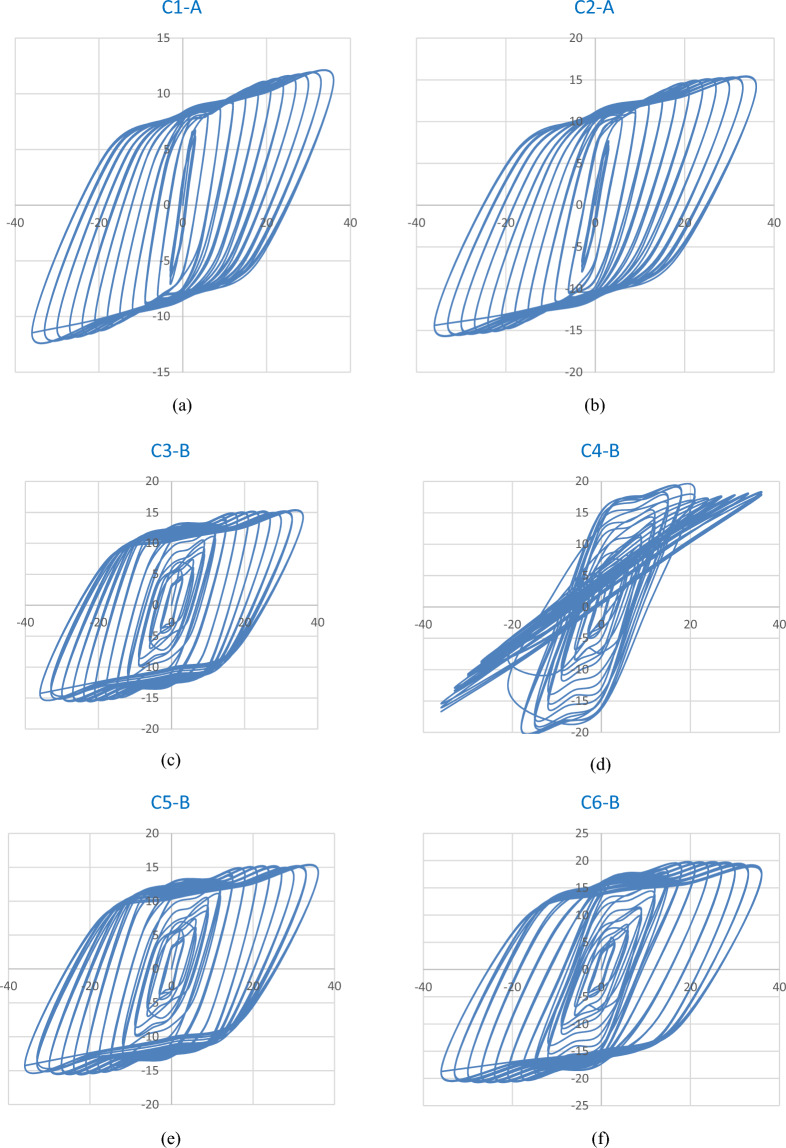
Hysteresis loops of UHPC column specimens reinforced with steel and basalt bars under cyclic lateral loading: (**a**) Specimen reinforced with 10 mm steel bars, showing the cyclic lateral load–displacement response. (**b**) Specimen reinforced with 12 mm steel bars, illustrating hysteretic behavior under increasing drift levels. (**c**) Specimen reinforced with 10 mm basalt bars, highlighting the hysteresis loop characteristics and stable energy dissipation. (**d**) Specimen reinforced with 12 mm basalt bars, demonstrating enhanced strength and reduced stiffness degradation. (**e**) Specimen reinforced with 10 mm basalt bars, showing consistent hysteretic response and effective crack control during cyclic loading. (**f**) Specimen reinforced with 12 mm basalt bars, presenting superior cyclic performance and larger energy dissipation capacity compared with steel-reinforced counterparts.

A clear visual agreement is observed between the experimentally captured and numerically predicted damage evolution for all specimens. This close correlation validates the robustness of the finite element model in simulating cracking behavior, deformation patterns, and ultimate failure mechanisms of UHPC columns reinforced with both steel and basalt bars Fig. 12.

### Analysis of numerical results

## Numerical envelope curves of UHPC columns reinforced with basalt bars under cyclic loading

**Fig. 13:**
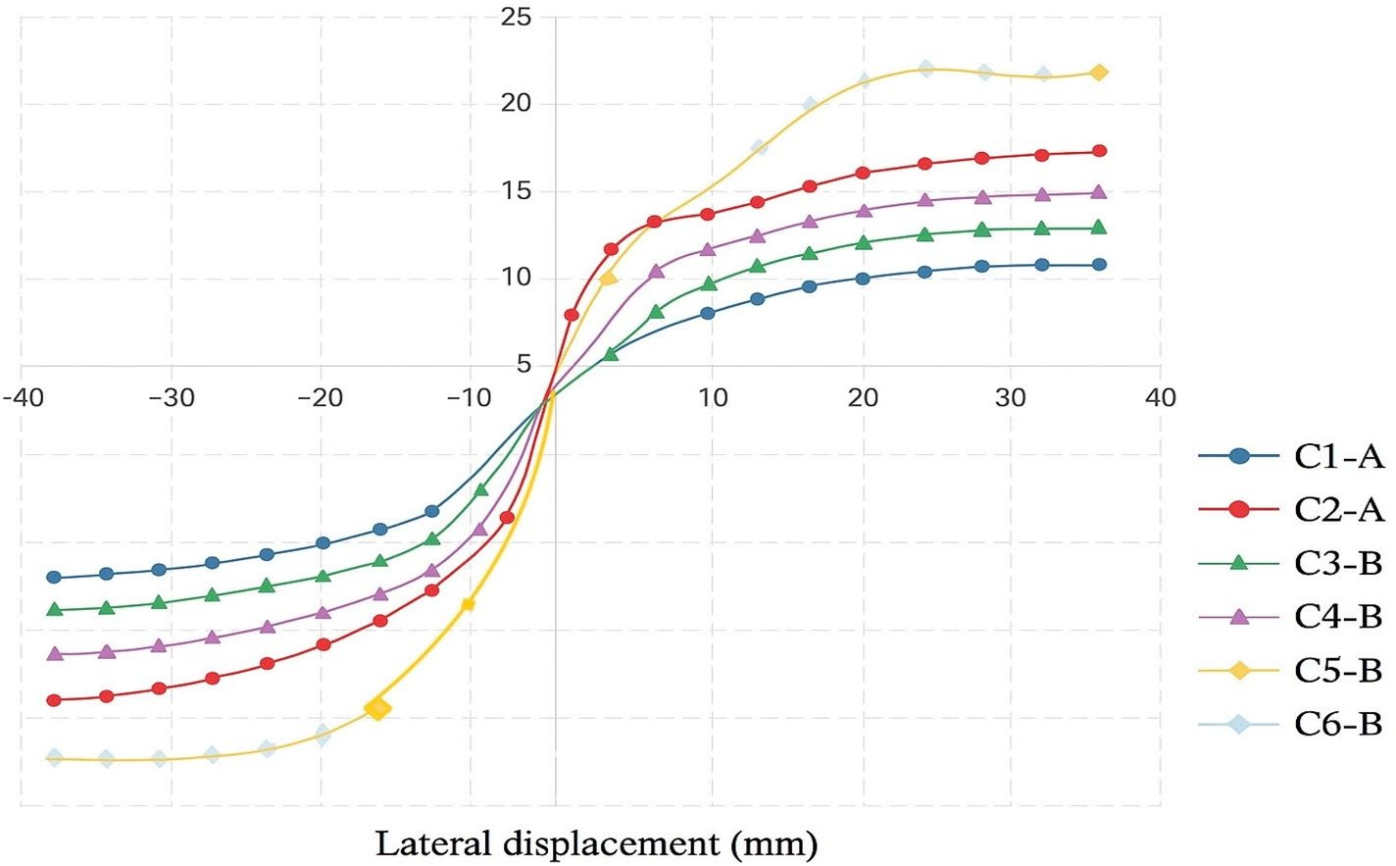
Lateral load–displacement envelope curves obtained from the numerical analysis using ABAQUS for the six simulated specimens with different reinforcement configurations and axial load ratios.

Finite element analysis was conducted using ABAQUS to simulate the cyclic behavior of six ultra-high-performance concrete (UHPC) columns reinforced with basalt fiber-reinforced polymer (BFRP) bars. For each numerical specimen, load–displacement hysteresis curves were generated, and the corresponding envelope curves were derived by connecting the peak points of each loading cycle. These envelope curves represent the maximum lateral load capacity and deformation characteristics of the UHPC columns under cyclic loading. The comparison of envelope responses among the six specimens highlights the influence of key parameters such as reinforcement configuration and axial load ratio. Moreover, the envelope curves derived serve as a basis for validating the finite element model against the experimental results, confirming the model’s ability to accurately capture the nonlinear cyclic behavior of UHPC columns (see Fig. 13).

## Comparison between experimental and finite element analysis results

This comparison between experimental outcomes and finite element analysis aims to verify the reliability and precision of the constructed 3D numerical models in simulating the structural performance of UHPC columns incorporating BFRP reinforcement. These models were examined based on key performance indicators, including peak lateral load capacity, maximum lateral displacement, and the overall crack distribution patterns observed during testing^58^.

### Numerical and experimental envelope curves for cyclic behavior of UHPC columns reinforced with steel and basalt bars

**Fig. 14:**
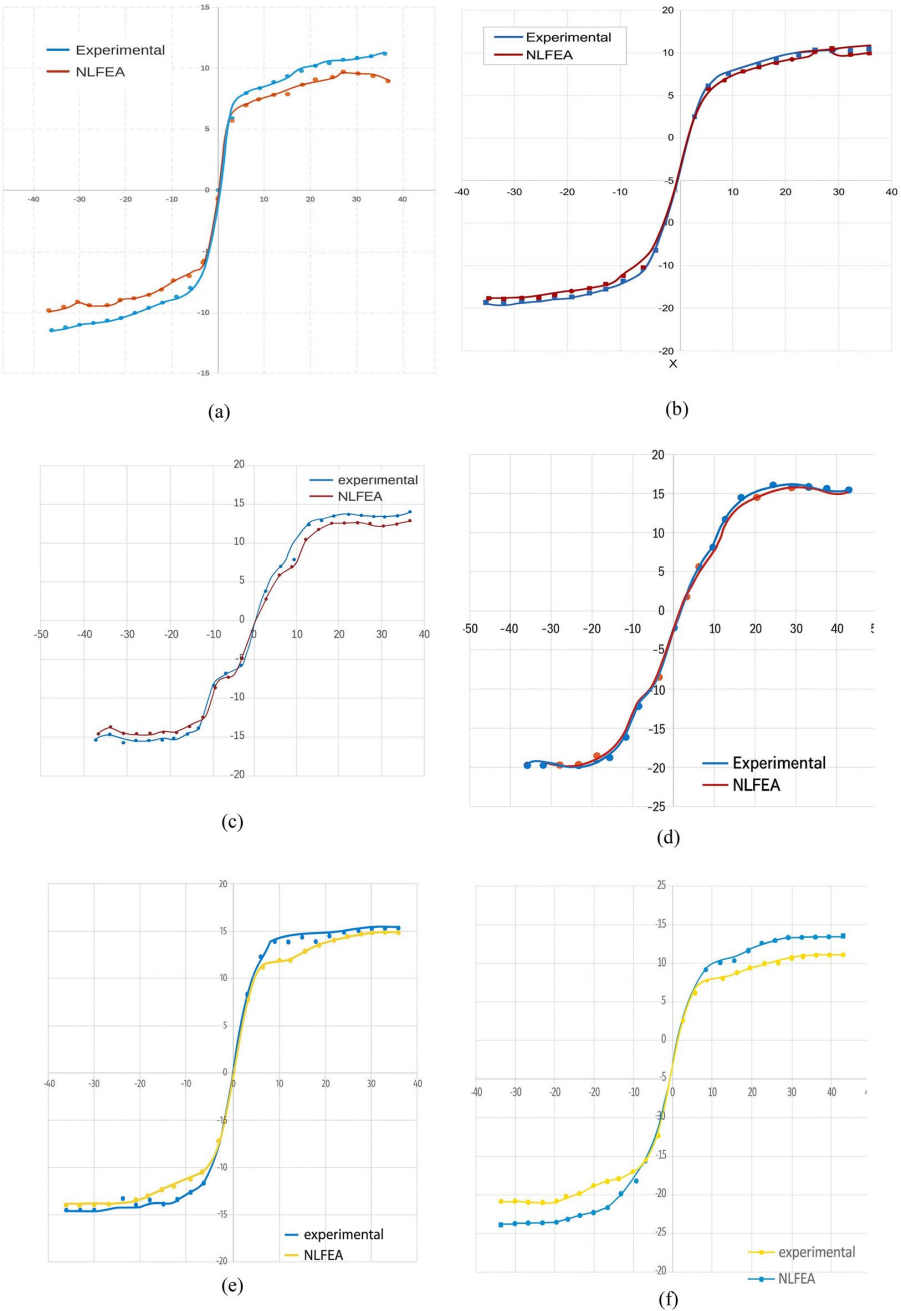
Comparison of experimental and numerical lateral load–displacement envelope curves for UHPC column specimens reinforced with steel and basalt bars under cyclic loading: (**a**) Specimen C1-A (steel, 10 mm bars). (**b**) Specimen C2-A (steel, 12 mm bars). (**c**) Specimen C3-B (basalt, 10 mm bars). (**d**) Specimen C4-B (basalt, 10 mm bars). (**e**) Specimen C5-B (basalt, 12 mm bars). (**f**) Specimen C6-B (basalt, 12 mm bars).

The envelope curves obtained from both experimental tests and finite element simulations using ABAQUS were compared for all six UHPC column specimens with different reinforcement types and sizes. The comparison demonstrates a strong correlation between the numerical and experimental results^59^, with similar percentages ranging approximately from 85 to 90%, confirming the reliability of the finite element model (see Fig. 14).

- Specimen C1-A, reinforced with 10 mm steel bars, showed excellent agreement between the experimental and numerical envelope curves, validating the model’s accuracy in capturing the structural behavior of steel-reinforced UHPC columns.
- For specimen C2-A, reinforced with 12 mm steel bars, the envelope curves from both approaches closely matched, further confirming the model’s capability to simulate columns with larger diameter steel reinforcement.
- Specimen C3-B, reinforced with 10 mm basalt fiber-reinforced polymer (BFRP) bars, exhibited envelope curves that closely resembled the experimental outcomes, indicating the finite element model’s suitability for simulating BFRP-reinforced columns.
- Similarly, specimen C4-B, reinforced with 10 mm basalt bars, showed an approximate similarity of 85% between numerical and experimental curves, reinforcing the model’s reliability for smaller diameter BFRP reinforcement.
- Specimen C5-B, reinforced with 12 mm basalt bars, achieved a high degree of similarity, emphasizing the model’s effectiveness in simulating larger diameter BFRP reinforcement behavior.
- Finally, specimen C6-B, also reinforced with 12 mm basalt bars, demonstrated a close match between the numerical simulations and experimental results, confirming the finite element model’s accuracy across all tested reinforcement configurations.
- This strong correlation between experimental and numerical envelope curves validates the finite element modeling approach in ABAQUS as an effective tool for predicting the nonlinear cyclic behavior of UHPC columns reinforced with both steel and basalt fiber bars under lateral cyclic loading^60^.

## Conclusions

Based on the comprehensive experimental program and numerical validation, the following concise, quantitative conclusions are drawn:

1. Quantitative performance. BFRP-reinforced UHPC columns achieved, on average, a 25.5% increase in ultimate lateral load capacity and exhibited ≈15% lower stiffness degradation at a 3% drift relative to steel-reinforced controls, confirming superior lateral strength and cyclic stability of the BFRP–UHPC system.
2. Failure mode. BFRP-reinforced columns showed a controlled brittle failure dominated by concrete crushing without bar buckling, unlike steel-reinforced specimens where yielding, cover spalling, and bar buckling governed the failure in the plastic-hinge zone.
3. Crack and deformation control. Use of BFRP longitudinal bars with BFRP stirrups reduced crack widths by 20–30% and improved post-cycle deformation recovery. The response is consistent with an effective passive confinement supplied by BFRP ties at high strain levels, which delays sudden crushing and stabilizes the hysteretic loops.
4. Material contribution. The UHPC matrix—optimized with silica fume and a polycarboxylate-based superplasticizer—produced a dense microstructure and compressive strengths of 85–100 MPa, supporting higher load capacity and durability while maintaining workable casting for slender members.
5. Design implications and real-world application.
6. Seismic regions and corrosive environments. BFRP–UHPC columns are promising for marine exposures, bridge piers subjected to de-icing salts, and coastal infrastructure, where corrosion resistance and cyclic robustness are critical.
7. Member proportioning. The demonstrated strength gain and reduced stiffness degradation support greater permissible slenderness or reduced cross-sectional areas while preserving safety margins, provided drift and detailing limits are respected.
8. Code development. Given the elastic–brittle nature of BFRP and the observed confinement effectiveness of BFRP stirrups in UHPC, future updates to ACI 440 and ECP 208^61^ should incorporate calibrated design parameters for UHPC–BFRP systems, including: (i) confinement efficiency factors for BFRP ties in UHPC; (ii) drift-dependent stiffness-degradation and energy-dissipation modifiers; and (iii) post-cracking stiffness provisions for envelope and backbone curve definition in nonlinear analysis.

These conclusions collectively indicate that partnering BFRP reinforcement with UHPC provides a structurally efficient, corrosion-resistant solution with quantifiable benefits in lateral strength, cracking control, and cyclic resilience, suitable for performance-based seismic design of critical infrastructure.

## Recommendations for future work:

The findings of this study provide valuable insights into the cyclic performance of UHPC columns reinforced with basalt fiber-reinforced polymer (BFRP) bars. However, further research is still required to expand the understanding and practical implementation of such systems. Based on the limitations and outcomes of the current investigation, the following directions are recommended for future studies:

1. Variation in reinforcement ratios:

Investigate the influence of different longitudinal and transverse BFRP reinforcement ratios on the strength, ductility, and energy dissipation capacity of UHPC columns under cyclic and seismic loading conditions.

2. Full-scale structural applications:

Extend the research to full-scale BFRP–UHPC structural members such as beams, bridge piers, and frame systems to validate laboratory-scale results and assess overall system performance.

3. Sustained and long-term loading effects:

Examine the impact of sustained axial loads, creep, and fatigue behavior over time to evaluate the long-term performance and serviceability of BFRP-reinforced UHPC structures.

4. Durability under harsh environmental conditions:

Study the combined effects of temperature variations, moisture, and chemical exposure on the bond durability between BFRP bars and UHPC, particularly in marine or corrosive environments.

5. Numerical modeling enhancements:

Improve finite element models by incorporating advanced damage plasticity formulations and bond–slip relationships specifically calibrated for BFRP–UHPC interfaces to enhance prediction accuracy.

6. Seismic design guidelines:

Develop practical design recommendations for BFRP–UHPC columns in accordance with international codes such as ACI 440 and ECP 208^61^, focusing on seismic performance parameters and safety factors.

## Data Availability

All data generated or analyzed during this study are included in this published article.
