# Correction: Structural performance of UHPC-columns reinforced with basalt bars under cyclic loading

**DOI:** 10.1038/s41598-026-40346-4

**Published:** 2026-02-17

**Authors:** Taha A. El-Sayed, Muhammed S. Fekry, Hossam E. Ahmed, Ali S. Shanour

**Affiliations:** Department of Civil Engineering, Shoubra Faculty of Engineering, Benha University, Cairo, Egypt

Correction to: *Scientific Reports* 10.1038/s41598-025-31316-3, published online 10 January 2026

The Author Contributions section in the original version of this Article was incomplete. As a result,

“Taha A. El-Sayed: supervised the student, prepared the research plan, shared in the theoretical work, and participated in writing and revised the final revision. Muhammed S. Fekry: wrote the article, carried out the experimental work, shared in the theoretical work and numerical modeling. Hossam E. Ahmed: supervised the student and revised the final revision. All authors read and approved of the final manuscript.”

now reads:

“Taha A. El-Sayed: supervised the student, prepared the research plan, shared in the theoretical work, and participated in writing and revised the final revision. Muhammed S. Fekry: wrote the article, carried out the experimental work, shared in the theoretical work and numerical modeling. Hossam E. Ahmed: supervised the student and revised the final revision. Ali S. Shanour: supervised the student, designed the experimental program methodology, performed formal analysis, and revised the final manuscript. All authors read and approved of the final manuscript.”

The original Article has been corrected.

